# NIR Spectrometric Approach for Geographical Origin Identification and Taste Related Compounds Content Prediction of Lushan Yunwu Tea

**DOI:** 10.3390/foods11192976

**Published:** 2022-09-23

**Authors:** Xiaoli Yan, Yujie Xie, Jianhua Chen, Tongji Yuan, Tuo Leng, Yi Chen, Jianhua Xie, Qiang Yu

**Affiliations:** 1 State Key Laboratory of Food Science and Technology, Nanchang University, Nanchang 330047, China; 2 Agriculture and Rural Bureau Agency of Lianxi District, Jiujiang 332005, China

**Keywords:** Lushan Yunwu tea, NIR, authenticity, taste-related indicators, prediction

## Abstract

Lushan Yunwu Tea is one of a unique Chinese tea series, and total polyphenols (TP), free amino acids (FAA), and polyphenols-to-amino acids ratio models (TP/FAA) represent its most important taste-related indicators. In this work, a feasibility study was proposed to simultaneously predict the authenticity identification and taste-related indicators of Lushan Yunwu tea, using near-infrared spectroscopy combined with multivariate analysis. Different waveband selections and spectral pre-processing methods were compared during the discriminant analysis (DA) and partial least squares (PLS) model-building process. The DA model achieved optimal performance in distinguishing Lushan Yunwu tea from other non-Lushan Yunwu teas, with a correct classification rate of up to 100%. The synergy interval partial least squares (siPLS) and backward interval partial least squares (biPLS) algorithms showed considerable advantages in improving the prediction performance of TP, FAA, and TP/FAA. The siPLS algorithms achieved the best prediction results for TP (R_P_ = 0.9407, RPD = 3.00), FAA (R_P_ = 0.9110, RPD = 2.21) and TP/FAA (R_P_ = 0.9377, RPD = 2.90). These results indicated that NIR spectroscopy was a useful and low-cost tool by which to offer definitive quantitative and qualitative analysis for Lushan Yunwu tea.

## 1. Introduction

Tea is one of the three greatest non-alcoholic beverages in the world, one that is preferred by many consumers and is particularly popular in China and Japan [1]. Tea contains many secondary metabolites, such as polyphenols, amino acids, polysaccharides, alkaloids, and terpenes, which are closely related to its quality and contribute to its rich taste, flavor, and health benefits [2]. It is generally known that the quality of tea is influenced by diverse factors, such as variety, original environment, plucking time, processing technology, and storage method [3,4]. Among them, the origin of tea is considered to be one of the key factors that directly determine its quality. There was an old Chinese saying, “Where there’s cloud and mist, there’s bound to be good tea”. It was beneath the natural wonder of tremendous clouds over Lushan Mountain that Lushan Yunwu tea was created. The mild and rainy climate is conducive to the accumulation of organic matter, such as amino acids and caffeine, so the tea has a delicate aroma and a mellow, fresh, and sweet flavor [5]. Lushan Yunwu tea was first produced in the Han Dynasty (202 BCE–220 CE), with a history of over 1800 years; since the Song Dynasty (960–1279), Lushan Yunwu was listed as the emperor’s and the imperial family’s tribute tea. In recent years, Lushan Yunwu tea was increasingly sought by tea tasters all over the world because of its unique geographical location and taste. Currently, Lushan Yunwu tea is included in the register of *Products of Geographical Indication* in China (GB/T 21003-2007).

Tea polyphenols (TP), total free amino acids (FAA), and their ratio of TP/FAA may be reliable taste quality indicators of tea flavor [5,6]. Generally, a high-quality green tea requires: (1) a proper concentration of TP to give the essential astringency; (2) a high concentration of FAA, which mainly contributes to the umami taste; and (3) an optimal balance between TP and FAA for a particular taste or pleasant flavor [1,6]. Research showed that the total phenolic contents of Lushan Yunwu tea were higher than those of other teas [7]. However, in recent years, many products of poor quality but similar in appearance to Lushan Yunwu tea have appeared in the market, pretending to be Lushan Yunwu tea, which has had a substantial impact on the sales price and market reputation of Lushan Yunwu tea. Therefore, the quality evaluation of Lushan Yunwu tea is an important topic, especially in preventing frauds that may affect the health of customers, or that may cause economic losses to producers and customers.

Traditionally, the authenticity identification and quality evaluation of tea mainly rely on the sensory evaluation of professional tea tasters. Although this method is relatively classic, the results are easily affected by factors such as the reviewer’s sensory organs and are highly subjective. The possible biases and changes in perception of the method make the results inaccurate and non-repeatable [8,9]. In addition, sensory evaluation cannot help us to know the contents of the main ingredients in tea. Ultraviolet-visible spectroscopy (UV-vis) [10,11], liquid chromatography mass spectrometry (LC-MS) [3,12], gas chromatograph-mass spectrometry (GC-MS) [13,14], and other technologies [8] can be used for the authenticity detection and quality determination of tea. These methods can accurately detect the chemical components that affect the quality of tea [9]. Nevertheless, most of these methods are prohibitively complicated, time–consuming, costly, and use a wide range of toxic solvents, so they are not suitable for the rapid testing of tea [9]. As an alternative, a variety of non-invasive detection technologies have been applied to rapidly evaluate the quality of tea, such as near-infrared spectroscopy technology (NIR) [15,16], hyperspectral imaging technology (HIS) [17], the E-nose [18], the E-tongue [19] and computer vision [20]. NIR technology is a mature testing technique with numerous advantages, such as being rapid, low-cost, nondestructive, and environmentally friendly [15,21,22]. Rapid identification methods for Anji white tea were developed using NIR spectroscopy and chemometric-class modeling techniques [21]. The same method has also been demonstrated to be an effective method for the identification of Darjeeling PGI tea [22]. Furthermore, NIR, when combined with multivariate calibration methods, has been shown to exhibit excellent analytical performance in predicting the main contents of tea leaves, including polyphenols, catechins, caffeine, and theanine [23,24]. In a recent study, Wang et al. used a micro NIR spectrometer to distinguish tea types (black, green, yellow, and oolong teas) and to predict catechins, caffeine, and theanine in four types of teas, and obtained satisfactory results [15]. However, there are few research reports on the simultaneous authenticity identification and taste quality assessments of tea, especially of Lushan Yunwu tea, a traditional and famous green tea.

Therefore, the main purpose of this work was to tackle the adulteration identification and quality prediction of Lushan Yunwu tea samples by the application of diffuse reflectance NIR spectra. The specific objectives of this study were: (1) to identify the authenticity of the origin of Lushan Yunwu tea; (2) to rapidly predict the taste quality indicators of Yunwu green tea (including TP, FAA, and TP/FAA) simultaneously by NIR; (3) to compare the performance of different spectral preprocessing methods and characteristic wavelength (or wavenumber) selection methods, in improving the accuracy of the prediction models.

## 2. Materials and Methods

### 2.1. Sample Preparation

Fifty-six Lushan Yunwu tea samples (LY) were obtained from various origins in Jiujiang, Jiangxi Province, China. Another 30 non-Lushan Yunwu tea samples (NLY) were collected in Fujian, Guangxi, and Sichuan provinces; these were processed using the same stir fixation as Lushan Yunwu tea. The details of the process and the representative images of tea samples are shown in Table S1 and Figure S1a of the Supplementary Materials. After being ground down for 30 s using a laboratory pulverizer (JYL-CO12, Joyoung Company Limited, Jinan, China) and passed through a 40-mesh sieve, all samples were individually packaged in light-proof sealed bags and stored at 4 °C before analysis.

### 2.2. Spectra Acquisition

Referring to the previous study [25] with slight modifications, the NIR spectra of tea samples were collected using a Nicolet 5700 FTIR spectrometer (Thermo Electron Corp., Madison, WI, USA). Briefly, about 1.0 g of tea powder was transferred into a standard quartz bottle (inner diameter, ∼10 mm) for testing. The NIR spectra were measured in the wavenumber range of 12,000–4000 cm^−1^ by using an average spectrum of 64 scans with a resolution of 8 cm^−1^.

### 2.3. Chemical Analysis

The TP content was determined using the Chinese standards method (GB/T8313-2018) with slight modifications. A 0.2 g sample of powdered tea was extracted with 5 mL of 70% (*v*/*v*) methanol solution at 70 °C for 10 min (stirred at 5 min intervals). The extract was left to cool to room temperature, then it was centrifuged at 3500 rpm for 10 min. The extraction process was repeated, and the supernatants were combined. The volume of the extract was adjusted to 10 mL with 70% (*v*/*v*) methanol and was filtered through a 0.45-μm microporous filter. The TP concentration was determined using the Folin–Ciocalteu reagent reaction, with a detection wavelength of 765 nm. The calibration standard was gallic acid and the results were expressed as gallic acid equivalents (GAE), in mg GAE/g dry matter.

The FAA contents were reference-measured using ninhydrin detection, as described in the Chinese standards method (GB/T8314-2013) with slight modifications. A mixture of tea powder (0.6 g) and 85 mL boiling water was placed in a boiling water bath for 45 min (stirred every 10 min). After that, the extract was immediately filtered under reduced pressure and washed with a little boiled water. Then, the filtered supernatant was adjusted to 100mL. First, 1 mL tea extract was mixed with 0.5 mL 1/15 mol/L phosphate buffer solution (pH 8.0) and 0.5 ml 2% ninhydrin (containing 0.8 mg/mL SnCl_2_·2H_2_O). The solution mixture was heated at 90 °C for 15 min, then the volume of the flask was adjusted to 25 mL after cooling down. The absorbance of the reaction solution was detected at 570 nm. The calibration standard was theanine and the results were expressed as theanine equivalents (TE), in mg TE/g of dry matter. TP/FAA was estimated as the TP content, divided by the FAA content.

### 2.4. Spectral Preprocessing

The tea samples were affected by external conditions during the NIR spectrum collection process, including sample state, particle size, baseline change, sample compactness difference, measurement environment difference, etc., which could cause various noises and information errors [26]. Therefore, before the multivariate statistical analysis took place, the spectral data needed to be preprocessed to reduce system noise and enhance the spectral features [27]. Multiplicative signal correction (MSC) is a type of widely applied preprocessing method for light-scattering correction, which is used to regulate the addition and multiplication effects in the spectrum on the foundation of different particle sizes [28]. Similar to MSC, the standard normal variate (SNV) is another mathematical transformation method for light-scattering correction; it is mainly used to eliminate the noise caused by optical path differences and uneven solid particles [29]. The first derivative (1st) and second derivative (2nd) can effectively eliminate the baseline shift and rotation error of the spectra [30]. However, the derivative transform promoted an emphasis on the level of noise while emphasizing the features of the spectral data, so it is often used in conjunction with a smoothing filter method, such as a Savitzky-Golay (SG) smoothing filter [25].

### 2.5. Multivariate Analysis

Tea samples were divided into two groups: three-quarters of the samples were used as the calibration set to develop DA and PLS models, and the remaining quarter of the samples was used as the prediction set to evaluate the performance of the models [31]. To avoid bias in sample selection, the sample data set was split following two principles: (1) regardless of whether the calibration set or prediction set was used, samples from every origin were included; (2) the samples containing extreme values for TP, FAA, and TP/FAA had to be included in the calibration set.

#### 2.5.1. Discrimination Analysis (DA)

The discrimination between Lushan Yunwu tea samples (LY) and non-Lushan Yunwu tea samples (NLY) was carried out using a discrimination analysis (DA) model. DA is a supervised classification technique with predefined groups. An unknown sample was assigned by calculating its Mahalanobis distance from the center of gravity of each group [25,32]. The greater the Mahalanobis distance between two given groups, the greater the spectral difference between them.

#### 2.5.2. Partial Least Squares (PLS)

The contents of TP, FAA, and TP/FAA in tea samples were predicted using the partial least squares (PLS) model. PLS projects the predictor variables and the observed variables into a new space and then captures several latent variables (LVs) that can represent the majority of raw information, using them to establish a linear regression model. The optimal number of factors was selected according to the residual sum of squares (PRESS) during the Leave One Out Cross-Validation (LOOCV) of the calibration models [32]. The factors where the first minimum value of PRESS appears is the optimal number of factors. The computed correlation coefficients in the calibration sets (R_C_) and prediction sets (R_P_), and their corresponding root mean square error of calibration (RMSEC) and prediction (RMSEP), along with the residual predictive deviation (RPD), were used to estimate and verify the accuracy of the developed models [33,34]. The RPD was calculated using the ratio between the standard deviation of the reference values and the RMSEP in the prediction set. In addition, the root mean square error of cross-validation (RMSECV) was used as a diagnostic value for model robustness. Good models have higher R (close to 1) and RPD (>2) but lower RMSE, and the RMSEP is lower than the RMSECV [35,36,37].

#### 2.5.3. Synergy Interval Partial Least Squares (siPLS)

The siPLS model is an effective characteristic variable selection algorithm proposed on the basis of interval partial least squares (iPLS) [38]. The entire spectra are equally divided into several equal intervals, then the different intervals are combined with each other. Then, the interval combination with the highest correlation coefficient and the lowest root mean square error of cross-validation (RMSECV) is selected. The combined intervals represent those spectral variables of the full spectra that are particularly relevant to the target parameter in the analysis.

#### 2.5.4. Backward Interval Partial Least Squares (biPLS)

The biPLS model is another characteristic variable selection method developed on the basis of iPLS [39]. The data set is divided into a given number of interval intervals of equal length and calculates RMSECV with each interval left out, leaving out one interval at a time [40]. After this process, the most relevant intervals are left behind.

### 2.6. Software

The spectral preprocessing and DA and PLS models were developed using the TQ Analyst (Thermo Nicolet Corporation, Madison, WI, USA). The siPLS and biPLS algorithms were carried out using the MATLAB R2020a software (MathWorks Inc., Natick, MA, USA).

## 3. Results and Discussion

### 3.1. Spectrum Description

**Figure 1:**
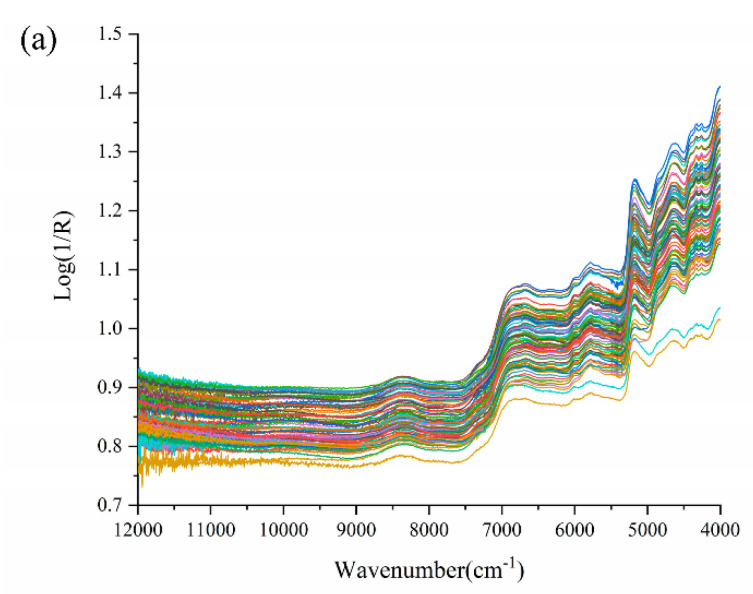

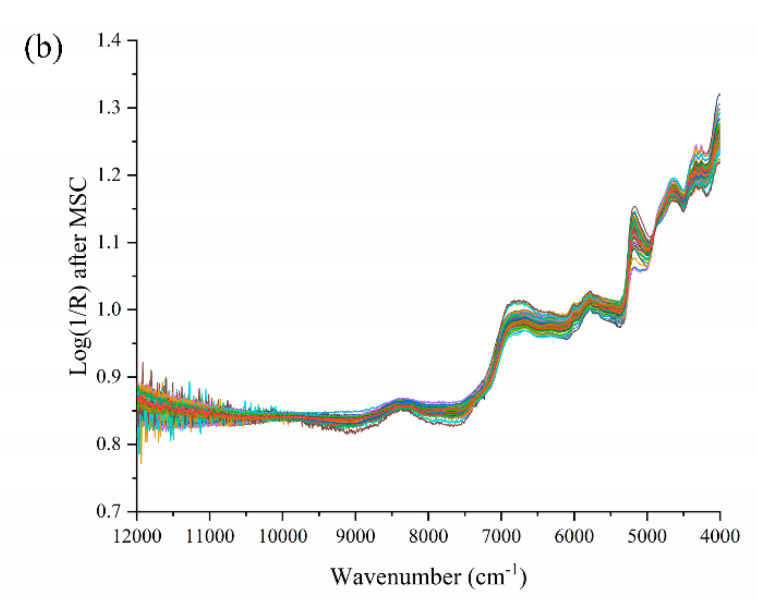
The spectra of all tea samples in the wavenumber range of 12,000–4000 cm^−1^. (**a**) original spectra (log 1/R); (**b**) spectra after pretreatment by multiplicative signal correction (MSC).

The raw NIR spectra of tea samples in the 12,000–4000 cm^−1^ frequency regions are shown in Figure 1a. The hydrogen-containing groups of organic matter in tea (such as C–H, O–H, S–H, and N–H, etc.) can produce multiple-frequency and combined-frequency absorptions in the near-infrared region, which are mainly related to the water and polysaccharides, polyphenols, amino acids, caffeine, and proteins in the tea [41]. The two weak peaks at 4258 cm^−1^ and 4327 cm^−1^ may be related to the second overtone of C–H bending and –CH_2_ bending, respectively [42]. The first overtone of C–H stretching was likely to appear near to 5780 cm^−1^. The absorption peak around 4650 cm^−1^ was related to the combination of N–H bending and C=O stretching [42]. Another absorption peak appeared at around 6700 cm^−1^, corresponding to the presence of the N–H group, which was attributed to free amino acids [41]. A wide and weak peak that appeared at 8600–8000 cm^−1^ may be associated with the C–H stretching of CH_2_ and CH_3_ groups, which was related to the presence of tea polyphenols [41]. In addition, since dry tea leaves generally contained 4–7% (*w*/*w*) of moisture, the absorption bands near 5160 cm^−1^ and 7000 cm^−1^ were also apparently displayed, which arose from the combination of O–H stretching and H-O-H deformation [25]. For some other wavenumber ranges (e.g., 12,000–10,000 cm^−1^), the spectrogram resembled noise and contained little spectral information on the active components. Figure 1a showed similar trends in all tea samples over the entire wavenumber range. Therefore, data preprocessing methods were generally used to eliminate noise and make the spectral features apparent after pretreatments, for example, in the case of the MSC (Figure 1b).

### 3.2. Authentication of Lushan Yunwu Tea

The representative images of tea samples are presented in Figure S1a of the Supplementary Materials, and it can be seen that it is impossible to clearly distinguish between the LY and NLY samples. The differences in geographic location cause differences in the chemical composition of the tea leaves. Figure S1b showed the descriptive statistics for the taste quality indicator of tea samples. It showed that LY samples contained more polyphenols and lower numbers of free amino acids than the NLY samples. However, no statistically significant differences were found in TP and FAA contents between LY and NLY samples (*p* > 0.05). Although TP/FAA showed significant differences between LY and NLY (*p* < 0.05), it was still difficult to distinguish LY and NLY samples solely by chemical composition analysis because of some overlapping ranges.

**Figure 2:**
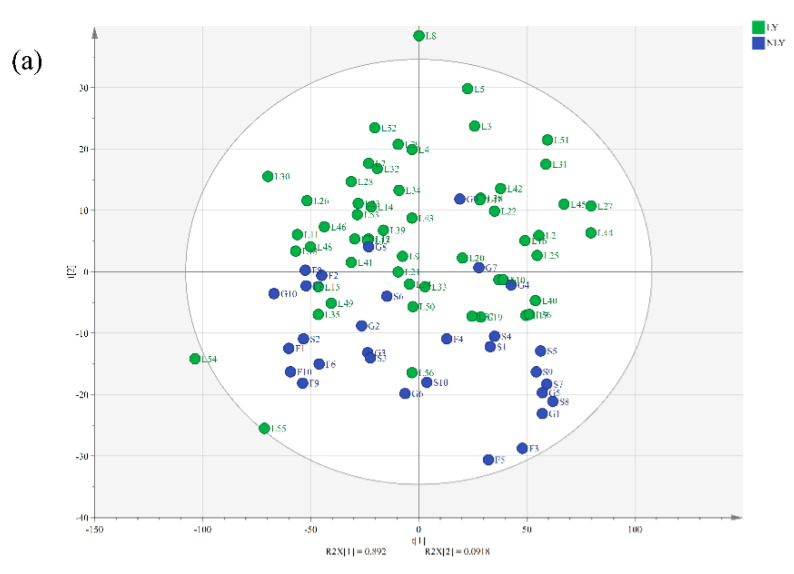

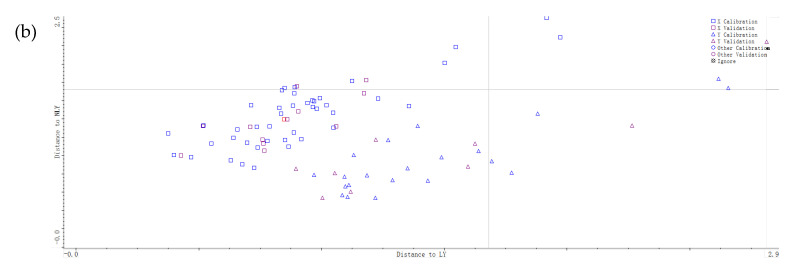
Classification result for Lushan Yunwu tea (LY) and non-Lushan Yunwu tea (NLY) discrimination. (**a**) Principal component analysis (PCA) model (green—LY, blue—NLY); (**b**) discrimination analysis (DA) model with MSC pretreatment in the wavenumber range of 9700–8600, 7400–6800, 5600–4000 cm^−1^ (□—LY, △—NLY).

In recent years, NIR spectroscopy has been widely used in the field of food authenticity identification because of its simplicity and convenience, so we used NIR technology to differentiate the samples of LY and NLY. To visualize the NIR data, a PCA model was used to analyze the natural distribution of the samples (Figure 2a). It was found that most of the LY and NLY samples could be distinguished in this unsupervised model. To further distinguish the two groups and to achieve authenticity prediction, the DA model was used.

Models with high classification accuracy were the most suitable for discrimination. It could be seen that models discriminating green tea origins with a degree of accuracy ranged from 62.79% to 100% when the DA models were developed using full-spectra data (Table 1). The first 9 PCs were utilized in the DA model, which covered the most variations (>62.49% of the total variance) contained in the spectral data. Interestingly, the application of spectral preprocessing did not improve the prediction accuracy and stability of the DA model. Generally, this result could be due to too many latent variables being included and may lead to overfitting of the calibration [25]. Thus, the wavenumber ranges needed to be selected in this study.

A spectral wavenumber range from 8000 to 4000 cm^−1^ was manually selected for the models (range 1), which has been reported to include most of the spectral information that reflects the metabolite components in tea [25]. Another manual selection of regions was conducted, based on the derivative transformed spectra, including 9700–8600 cm^−1^, 7400–6800 cm^−1^, and 5600–4000 cm^−1^ (range 2). The performance of the DA models, established with different selected wavenumber ranges and preprocessing methods, is shown in Table 1.

Compared to the DA models built using full spectral wavenumbers, DA models with manual wavenumber selections were more efficient in terms of accuracy and robustness, especially when based on the wavenumber range 2. The DA models with MSC and SNV pretreatment performed best using the wavenumber ranges of 9700–8600, 7400–6800, and 5600–4000 cm^−1^. The Mahalanobis distance plot of every sample to the center of gravity of two classes, established by a DA model with MSC pretreatment, is shown in Figure 2b. The LY samples were completely separated from the NLY tea group, which indicated that NIR, combined with the DA model, may be a potential method for the authenticity discrimination of Lushan Yunwu tea with sufficient sensitivity. As far as we know, this was the first time that near-infrared technology was used to identify and predict the authenticity of Lushan Yunwu tea and obtained satisfactory results. It was worth noting that the derivative transform pretreatment did not play a helpful role in optimizing this model, which may be because the derivative transform promoted an emphasis on the level of noise while emphasizing the features of the spectral data. 

### 3.3. PLS Models for TP, FAA, TP/FAA Prediction

TP contributes to the essential astringency in green tea, while FAA mainly contributes to the mellowness and umami of green tea; these two components are often used as important indicators to evaluate the quality of tea [23,43]. TP/AA reflects the relationship between the umami taste and astringency and is used to reflect the quality of tea in previous studies [33]. In this study, the three indicators of TP, FAA, and TP/FAA were predicted using NIR technology. PLS models were developed with raw and preprocessed spectral data to predict TP, FAA, and TP/FAA in different tea samples (Table 2). For TP prediction, the raw-PLS model with the full-spectra data achieved an acceptable performance, with R_C_ = 0.9303 and RMSEC = 8.05 in the calibration set, and R_P_ = 0.8546, RMSEP = 14.2 in the prediction set. Compared with the performance of the original data, the performance of the models after MSC and SNV spectral pretreatment had been improved, with higher R_P_ and lower RMSEP. However, other methods of spectral preprocessing did not play an effective role. In the case of FAA, the performance of all PLS models was relatively poor (R_C_, R_P_ < 0.9), indicating that all PLS models based on full-spectra data were not ideal for predicting free amino acid content. For TP/FAA, the MSC-PLS model performed best, with the highest R_P_ = 0.8430, RPD = 1.88 and the lowest RMSEP = 0.593. It showed that the prediction of TP/FAA by the PLS model, based on the full-spectra data, was also unsatisfactory. As in the DA models, the derivative transformation preprocessing did not play an effective role in the optimization of PLS models for prediction in tea samples. Compared with polyphenols, the values of amino acid contents were much lower; this may explain why the prediction accuracy of FAA and TP/FAA was low [15]. In addition, the full spectrum contained redundant information that had nothing to do with amino acids, which could reduce the prediction accuracy [15]. Therefore, a variable filter was needed to optimize the PLS models.

### 3.4. Variables Selection and PLS Models Optimization

#### 3.4.1. Prediction Models Based on the Manual Selected Wavenumber Range

In order to remove redundant information that had nothing to do with the tea flavor components, the PLS models were optimized by manually selected wavenumbers. As noted in Section 3.2, two manually selected wavenumber ranges were applied (Table 2). The models established with manually selected wavenumber ranges achieved better results than those that were performed based on the full-spectra region. However, regardless of the wavenumber range and the spectral preprocessing method that was used, the PLS models concerning FAA did not achieve a good performance, with R_P_ values lower than 0.9 and RPD values lower than 2.0. These results indicated that the wavenumber selection range needs to be optimized further.

#### 3.4.2. Prediction Models Based on the siPLS and biPLS Algorithms

**Figure 3:**
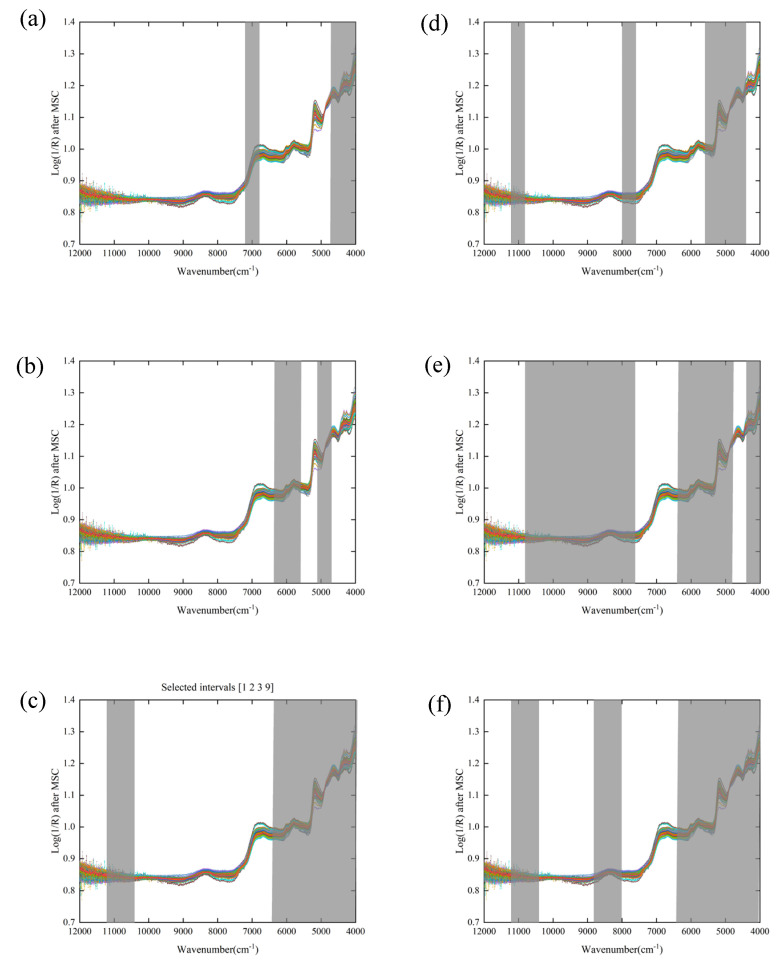
The optimization of spectral intervals, developed by siPLS and biPLS algorithm for quality compounds: (**a**) siPLS for TP; (**b**) siPLS for FAA; (**c**) siPLS for TP/FAA; (**d**) biPLS for TP; (**e**) biPLS for FAA; (**f**) biPLS for TP/FAA.

The siPLS and biPLS algorithms were used to select wavenumber variables that were closely related to the tea taste quality indicators. The optimal model results for the TP, FAA, and TP/FAA of tea can be seen in Table 3. Firstly, the full-spectra data were equally split into 10 or 20 sub-intervals, as in the siPLS modeling. The siPLS algorithm selected optimal sub-interval combinations for TP, FAA, and TP/FAA with the lowest RMSECV values (Figure 3a–c). The wavenumber ranges of 7204.87–6807.60 cm^−1^ and 4798.10–3999.70 cm^−1^ that were selected for TP corresponded to the C–H stretching and C–H deformation combinations [44]. The optimal sub-intervals were 6402.61–5604.21 and 5199.23–4801.96 cm^−1^ for FAA and 11,200.71–10,406.17, 6402.61–3999.70 cm^−1^ for TP/FAA. The siPLS algorithm not only reduced the amount of wavenumber data required for modeling (TP (312), FAA (312), TP/FAA (831)) but also greatly improved the accuracy of the prediction model, especially in terms of FAA and TP/FAA prediction (Table 3 and Figure S2 in the Supplementary Materials). After using the siPLS algorithm to filter wavenumbers, the R_P_ for TP, FAA, and TP/FAA prediction increased from 0.8546, 0.8490, and 0.8089 to 0.9407, 0.9110, and 0.9377, and the RPD values improved from 1.91, 1,62, and 1.73 to 3.00, 2.21, and 2.90, respectively. These proved the effectiveness of the siPLS model in selecting a few spectral variables from the optimized combination of sub-intervals, to determine the target of interest [45].

Compared with siPLS, biPLS is also a method for the joint modeling of several sub-intervals for screening correlations, but the biPLS algorithm has the advantage of filtering only the characteristic wavenumber sub-intervals in the backward direction; it rejects the sub-intervals with the worst correlation each time [40]. Removed permanently, the last sub-intervals that are left are the spectral variables that have the highest correlation with the tea flavor components. The performance of biPLS models for TP, FAA, and TP/FAA are shown in Table 3 and Figure S3 in the Supplementary Materials. For TP prediction, the biPLS selected 519 relevant characteristic variables from 2075 variables (Figure 3d). The R_P_ and RPD values for TP prediction increased from 0.8546 and 1.91 in the original PLS model to 0.9508 and 3.26 in the biPLS model, respectively. For FAA prediction, biPLS selected the wavenumber ranges of 10,807.3–7609.85 cm^−1^, 6402.61–4801.96 cm^−1^, 4396.97–3999.7 cm^−1^ (Figure 3e); the model prediction accuracy was significantly higher than the original PLS model prediction accuracy. The R_P_ (from 0.7619 to 0.9492) and the RPD (from 1.62 to 2.07) increased, and the RMSEP decreased from 6.79 to 5.31. The biPLS algorithm selected 1013 relevant variables to predict the TP/FAA (Figure 3f). Compared with the original PLS model, based on the full-spectra data, the correlation coefficient R_P_ of the model in the prediction set was significantly improved (from 0.8089 to 0.9303). However, the RMSECV values of the biPLS models were all greater than those in the corresponding siPLS models, indicating that the siPLS models were more robust for predicting TP, FAA, and TP/FAA.

The spectral regions selected by the siPLS and biPLS methods had an abundant overlap. The region that was referred to by the wavenumbers of 4798.10–3999.70 cm^−1^ was selected for TP in both algorithms, which included a combination of C–H stretching and C–H deformation, and the second overtone of C–H deformation. This spectral region was associated with the reported presence of tea polyphenols and caffeine and contained an important spectral region for identifying special-grade green tea from other grades of green tea [46]. Two regions, 6402.61–5604.21 and 5199.23–4801.96 cm^−1^, were important for building the FAA prediction model. The 5199.23–4801.96 cm^−1^ region reflected the –NH group co-frequency information and 6402.61–5604.21 cm^−1^ contained overtones of both the –CH_2_ (5750 cm^−1^) and –CH_3_ (5800 cm^−1^) [47], which were related to the presence of free amino acids. One finding of note was that the model performance of FAA was worse than that of TP and TP/FAA in all PLS models. Wang et al. also found that the prediction performance of the PLS model for the theanine content in tea was not satisfactory [15]. This may be because the relationship between free amino acids and near-infrared spectroscopy may be non-linear; the non-linear algorithms may be more appropriate to improve the model prediction accuracy of NIR spectroscopy for free amino acids in future studies.

Compared with the PLS model established by the full-spectra data or manually selected wavenumber range, the prediction performance of the PLS model, established after using the siPLS and biPLS algorithms to select the characteristic wavenumber range, had been greatly improved, especially for FAA and TP/FAA prediction. As observed in this study, the combination of siPLS and biPLS with NIR could greatly improve the prediction of flavor components in Yunwu green tea.

## 4. Conclusions

In this study, the general prediction models for TP, FAA, and TP/FAA in Lushan Yunwu tea and non-Lushan Yunwu tea were developed using NIR, combined with multivariate analysis. The NIR spectra of LY and NLY could be distinguished according to geographical origin, while the correct discrimination rate of DA models could reach 100%. The effects of different spectral preprocessing and wavenumber selection on the performance of PLS prediction models for TP, FAA, and TP/FAA were compared. The siPLS model achieved satisfactory performance for TP and TP/FAA predictions, with RP values higher than 0.9 and RPD values higher than 2.0. The results showed that the combination of NIR and intelligent variable selection algorithms (such as biPLS and siPLS) could achieve the rapid prediction of taste quality indicators in tea. Therefore, NIR spectroscopy may be a green analysis tool that can predict the taste quality indicators in tea while identifying the authenticity of the tea.

## Figures and Tables

**Table 1:** Performance of DA models with different spectral preprocessing approaches.

Wavenumber Range	Pretreatment Methods	Factors	% of Variability Described	No. of Incorrectly Classified Samples		% of Samples Correctly Classified
				LY (*n* = 56)	NLY (*n* = 30)	
Full wavenumbers (12,000–4000 cm^−1^)	None	9	99.93	0	0	100
	MSC	9	97.01	0	0	100
	SNV	9	96.93	0	0	100
	1st derivative	9	62.49	31	0	63.95
	2nd derivative	9	63.44	30	1	63.95
	MSC + 1st + SG filter (7, 3)	9	75.34	31	1	62.79
	SNV + 1st + SG filter (7, 3)	9	75.20	31	1	62.79
Range 1 (8000–4000 cm^−1^)	None	9	99.99	0	4	95.35
	MSC	9	99.70	0	1	98.84
	SNV	9	99.65	0	1	98.84
	1st derivative	9	90.31	2	2	95.35
	2nd derivative	9	91.27	24	2	69.77
	MSC + 1st + SG filter (7, 3)	9	90.23	0	3	96.51
	SNV + 1st + SG filter (7, 3)	9	90.21	0	3	96.51
Range 2 (9700–8600 + 7400–6800 + 5600–4000 cm^−1^)	None	9	99.99	3	2	94.19
	**MSC**	**9**	**99.42**	**0**	**0**	**100**
	**SNV**	**9**	**99.41**	**0**	**0**	**100**
	1st derivative	9	86.22	3	3	93.02
	2nd derivative	9	87.33	29	1	65.12
	MSC + 1st + SG filter (7, 3)	9	86.92	2	3	94.19
	SNV + 1st + SG filter (7, 3)	9	86.89	2	3	94.19

Abbreviations: MSC, multiplicative signal correction; SNV, standard normal variate; 1st, first derivative; 2nd, second derivative; SG, Savitzky-Golay smoothing.

**Table 2:** The performance of partial least squares (PLS) models with different spectral preprocessing approaches for the prediction of total polyphenols content (TP), free amino acids content (FAA), and polyphenols-to-amino acids ratio (TP/FAA), based on different wavenumber ranges.

Wavenumber Range	Pretreatment Methods	TP							FAA							TP/FAA						
		Factors	Calibration Set			Prediction Set			Factors	Calibration Set			Prediction Set			Factors	Calibration Set			Prediction Set		
			R_C_	RMSEC	RMSECV	R_P_	RMSEP	RPD		R_C_	RMSEC	RMSECV	R_P_	RMSEP	RPD		R_C_	RMSEC	RMSECV	R_P_	RMSEP	RPD
Full wavenumbers (12,000–4000 cm^−1^)	None	8	0.9303	8.05	13.4	0.8546	14.2	1.91	6	0.7619	6.08	7.74	0.8490	6.79	1.62	8	0.9356	0.310	0.553	0.8089	0.645	1.73
	MSC	7	0.9167	8.77	13.8	0.9086	11.5	2.36	2	0.4881	8.20	8.83	0.4967	9.62	1.14	7	0.9119	0.360	0.686	0.8430	0.593	1.88
	SNV	7	0.9184	8.68	13.8	0.9073	11.6	2.34	2	0.4900	8.19	8.82	0.4989	9.61	1.14	7	0.9141	0.356	0.692	0.8352	0.603	1.85
	1st derivative	3	0.8940	9.83	20.6	0.7821	19.9	1.36	1	0.5134	8.06	10.0	0.5803	9.66	1.14	4	0.9612	0.242	0.86	0.7477	0.782	1.43
	2nd derivative	2	0.7237	15.10	21.3	0.3379	25.6	1.06	3	0.8715	4.61	9.88	0.0681	11.1	0.99	3	0.8970	0.388	0.869	0.3258	1.04	1.07
	MSC + 1st + SG filter (7, 3)	4	0.8990	9.61	20.6	0.8101	18.7	1.45	1	0.3511	8.79	9.70	0.4309	10.5	1.05	1	0.4057	0.802	0.896	0.7797	1.01	1.10
	SNV + 1st + SG filter (7, 3)	4	0.8994	9.59	20.6	0.8099	19.7	1.38	1	0.3521	8.79	9.70	0.4340	10.5	1.05	1	0.4066	0.802	0.896	0.7806	1.01	1.10
Range 1 (8000–4000 cm^−1^)	None	9	0.9085	9.17	12.3	0.8666	13.6	2.00	10	0.8687	4.65	6.78	0.8507	6.84	1.60	10	0.9195	0.345	0.514	0.8363	0.618	1.80
	MSC	8	0.9054	9.31	12.0	0.9028	11.5	2.36	6	0.8559	4.86	7.08	0.8762	6.23	1.76	8	0.8980	0.386	0.55	0.8739	0.545	2.05
	SNV	7	0.9021	9.47	12.2	0.8590	13.8	1.97	8	0.8520	4.92	7.24	0.8822	6.16	1.78	8	0.8974	0.387	0.561	0.8652	0.559	1.99
	1st derivative	6	0.9778	4.59	13.9	0.8958	12.6	2.15	6	0.9649	2.46	7.82	0.7796	7.85	1.40	5	0.9787	0.180	0.591	0.8312	0.656	1.70
	2nd derivative	5	0.9847	3.83	21.4	0.2931	25.3	1.07	2	0.6833	6.86	9.69	0.6390	9.47	1.16	6	0.9957	0.081	0.884	0.5921	0.945	1.18
	MSC + 1st + SG filter (7, 3)	5	0.9525	6.68	13.7	0.9264	11.2	2.42	6	0.9759	2.05	7.22	0.8480	6.96	1.58	7	0.9929	0.104	0.562	0.8676	0.610	1.83
	SNV + 1st + SG filter (7, 3)	5	0.9527	6.67	13.8	0.9261	11.2	2.42	6	0.9759	2.05	7.22	0.8461	7.00	1.57	7	0.9931	0.103	0.561	0.8667	0.612	1.82
Range 2 (9700–8600 + 7400–6800 + 5600–4000 cm^−1^)	None	9	0.9199	8.60	12.5	0.8561	13.9	1.95	10	0.8793	4.47	6.62	0.8648	6.74	1.63	10	0.9371	0.306	0.505	0.9264	0.466	2.39
	MSC	7	0.9046	9.35	12.4	0.8953	11.8	2.30	9	0.9260	3.54	7.28	0.8079	7.39	1.49	8	0.9238	0.336	0.566	0.8551	0.590	1.89
	SNV	5	0.8655	11.00	13.6	0.8782	12.9	2.10	9	0.8874	4.33	7.42	0.8315	6.92	1.59	8	0.9120	0.360	0.561	0.8905	0.524	2.13
	1st derivative	5	0.9427	7.32	16.7	0.9111	12.7	2.14	5	0.9346	3.34	9.14	0.7021	8.39	1.31	6	0.9765	0.189	0.723	0.7915	0.709	1.57
	2nd derivative	2	0.6324	17.00	21.9	0.5005	24.4	1.11	2	0.5953	7.54	9.95	0.5813	9.97	1.10	6	0.9900	0.124	0.951	0.5868	0.943	1.18
	MSC + 1st + SG filter (7, 3)	5	0.9426	7.33	16.8	0.9118	12.2	2.23	6	0.9689	2.32	8.46	0.7297	8.11	1.35	6	0.9728	0.203	0.731	0.8218	0.657	1.70
	SNV + 1st + SG filter (7, 3)	5	0.9425	7.33	16.8	0.9115	12.2	2.23	6	0.9724	2.19	8.52	0.7261	8.13	1.35	6	0.9713	0.209	0.737	0.8220	0.658	1.69

Abbreviations: R_C_, correlation coefficients of calibration; (R_P_) correlation coefficients of prediction; RMSEC, root mean square error of calibration; RMSECV, root mean square error of cross validation; RMSEP, root mean square error of prediction; RPD, residual predictive deviation.

**Table 3:** The performance of PLS models, with different characteristic wavenumber selection procedures for the prediction of polyphenols, free amino acids content, and the polyphenols-to-amino acids ratio.

Methods	Tea Polyphenols Content								Free Amino Acids Content								TP/AA							
	Variables	Factors	Calibration Set			Prediction Set			Variables	Factors	Calibration Set			Prediction Set			Variables	Factors	Calibration Set			Prediction Set		
			R_C_	RMSEC	RMSECV	R_P_	RMSEP	RPD			R_C_	RMSEC	RMSECV	R_P_	RMSEP	RPD			R_C_	RMSEC	RMSECV	R_P_	RMSEP	RPD
Full	2075	8	0.9303	8.05	13.4	0.8546	14.2	1.91	2075	6	0.7619	6.08	7.74	0.8490	6.79	1.62	2075	8	0.9356	0.31	0.553	0.8089	0.645	1.73
siPLS	312	9	0.9344	7.82	12.0	0.9407	9.04	3.00	312	9	0.9103	3.89	6.3	0.9110	4.96	2.21	831	9	0.9641	0.233	0.466	0.9377	0.385	2.90
biPLS	519	7	0.9125	8.79	13.5	0.9508	8.33	3.26	1454	9	0.9492	2.95	7.2	0.9199	5.31	2.07	1013	9	0.9420	0.295	0.645	0.9303	0.437	2.55

Abbreviations: RMSEC, root mean square error of calibration; RMSECV, root mean square error of cross validation; RMSEP, root mean square error of prediction; RPD, residual predictive deviation.

## Data Availability

Not applicable.

## Supplementary Materials

The following supporting information can be downloaded at: https://www.mdpi.com/article/10.3390/foods11192976/s1, Figure S1. Boxplots of TP, FAA, and TP/FAA contents in the LY and NLY samples; Figure S2. Correlation between the measured and predicted values of siPLS models; Figure S3. Correlation between the measured and predicted values of biPLS models; Table S1. A summary of the tested samples.
