# Supplementary material for: NIR Spectrometric Approach for Geographical Origin Identification and Taste Related Compounds Content Prediction of Lushan Yunwu Tea
Source: Foods. 2022 Sep 23;11(19):2976. doi: 10.3390/foods11192976 (PMC9563823; doi:10.3390/foods11192976)
Supplement: Supplementary file 1 [file foods-11-02976-s001.zip › foods-1920899-supplementary.pdf]

Supplementary materials

Article

NIR spectrometric approach for geographical origin identification and taste related compounds content prediction of Lushan Yunwu Tea

Xiaoli Yan[1], Yujie Xie[1], Jianhua Chen [2], Tongji Yuan [1], Tuo Leng [1], Yi Chen[1,*], Jianhua Xie[1], Qiang Yua [1]

1 State Key Laboratory of Food Science and Technology, Nanchang University, Nanchang 330047, China
2 Agriculture and Rural Bureau Agency of Lianxi District, Jiujiang 332005, China
* Correspondence: Correspondence: chenyi-417@163.com; Tel./Fax: +86-0791-88304449

Abstract: Lushan Yunwu Tea is one of the unique Chinese tea series, and total polyphenols (TP), free amino acids (FAA), and polyphenols-to-amino acids ratio models (TP/FAA) are its important taste related indicators. In this work, a feasibility study was proposed to simultaneously predict the authenticity identification and taste related indicators of Lushan Yunwu tea using near-infrared spectroscopy combined with multivariate analysis. Different waveband selections and spectral pre-processing methods were compared during the discriminant analysis (DA) and partial least square (PLS) models building process. DA model achieved optimal performance in distinguishing Lushan Yunwu tea from other non-Lushan Yunwu tea with a correct classification rate up to 100%. The synergy interval partial least square (siPLS) and backward interval partial least square (biPLS) algorithms showed considerable advantages in improving the prediction performance of TP, FAA, and TP/FAA. The siPLS algorithms achieved the best prediction results for TP (RP = 0.9407, RPD = 3.00), FAA (RP = 0.9110, RPD = 2.21) and TP/FAA (RP = 0.9377, RPD = 2.90). These results indicated that NIR spectroscopy was a useful and low-cost tool to offer definitive quantitative and qualitative analysis for Lushan Yunwu Tea.

Keywords: Lushan Yunwu Tea, NIR, authenticity, taste related indicators, prediction

Table S1. A summary of tested samples.

Sample No.	Origin	Logo	Harvesting Season	Fixation	Category
L1-L7	Bailu, Jiujiang	Yunwu Tea	Spring	Stir fixation	LY
L8	Donggushan, Jiujiang	Yunwu Tea	Spring	Stir fixation	LY
L9-L18	Gaolong, Jiujiang	Yunwu Tea	Spring	Stir fixation	LY
L19-L20, L22-L23, L46-L47	Lushan Mountain, Jiujiang	Yunwu Tea	Spring	Stir fixation	LY
L21	Lianhua, Jiujiang	Yunwu Tea	Spring	Stir fixation	LY
L24-L26	Minshan, Jiujiang	Yunwu Tea	Spring	Stir fixation	LY
L27-L34	Saiyang, Jiujiang	Yunwu Tea	Spring	Stir fixation	LY
L35-L39	Tongyuan, Jiujiang	Yunwu Tea	Spring	Stir fixation	LY
L40-L45	Weijia, Jiujiang	Yunwu Tea	Spring	Stir fixation	LY
L48-L50	Chaisang, Jiujiang	Yunwu Tea	Spring	Stir fixation	LY
L51-L56	Yujiahe, Jiujiang	Yunwu Tea	Spring	Stir fixation	LY
F1-F10	Fujian	Yunwu Tea	Spring	Stir fixation	NLY
G1-G10	Guangxi	Yunwu Tea	Spring	Stir fixation	NLY
S1-S10	Sichuan	Yunwu Tea	Spring	Stir fixation	NLY

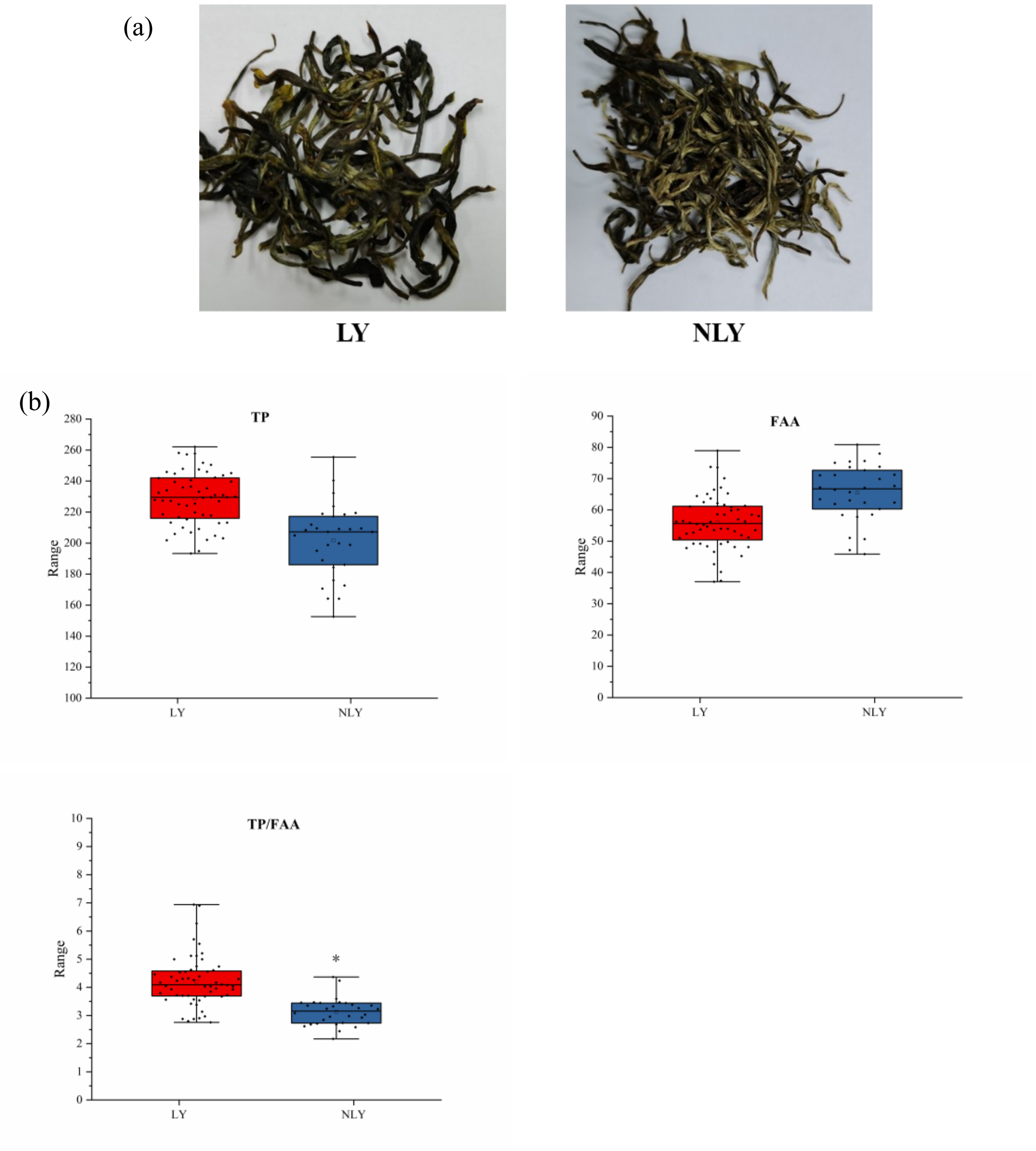

Figure S1. (a) The representative images of LY and NLY samples; (b) boxplots of TP, FAA and TP/FAA contents in the LY and NLY samples. Note: “*” indicated a significant difference between the two groups ($p<0.05$).

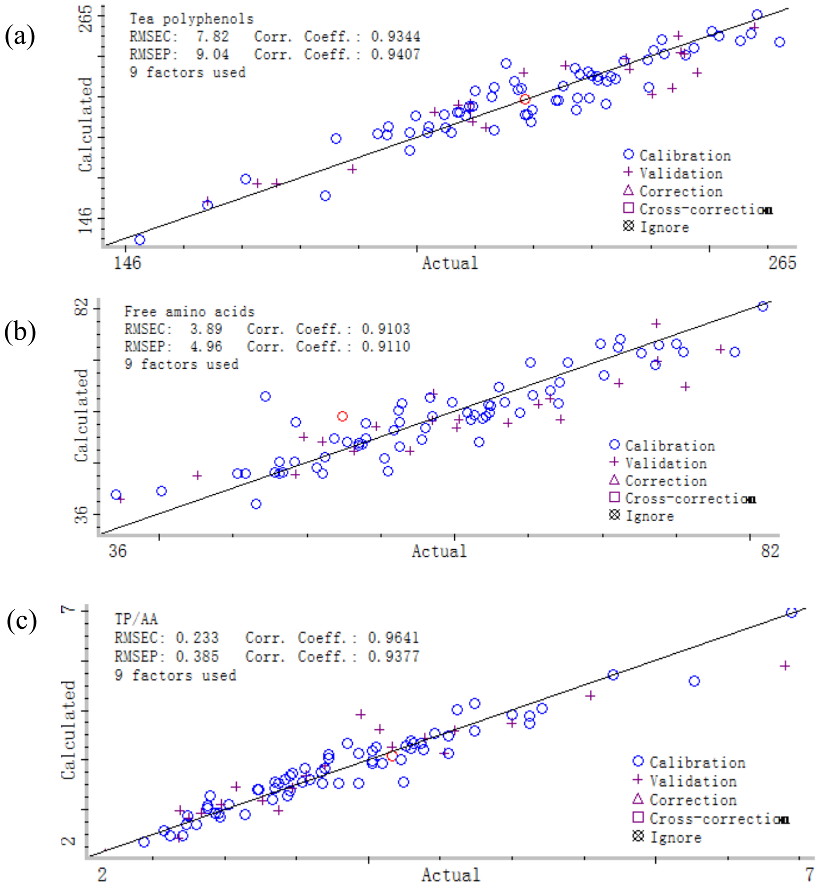

Figure S2. Correlation between the measured and predicted values of siPLS models. (a) TP; (b) FAA; (c) TP/FAA.

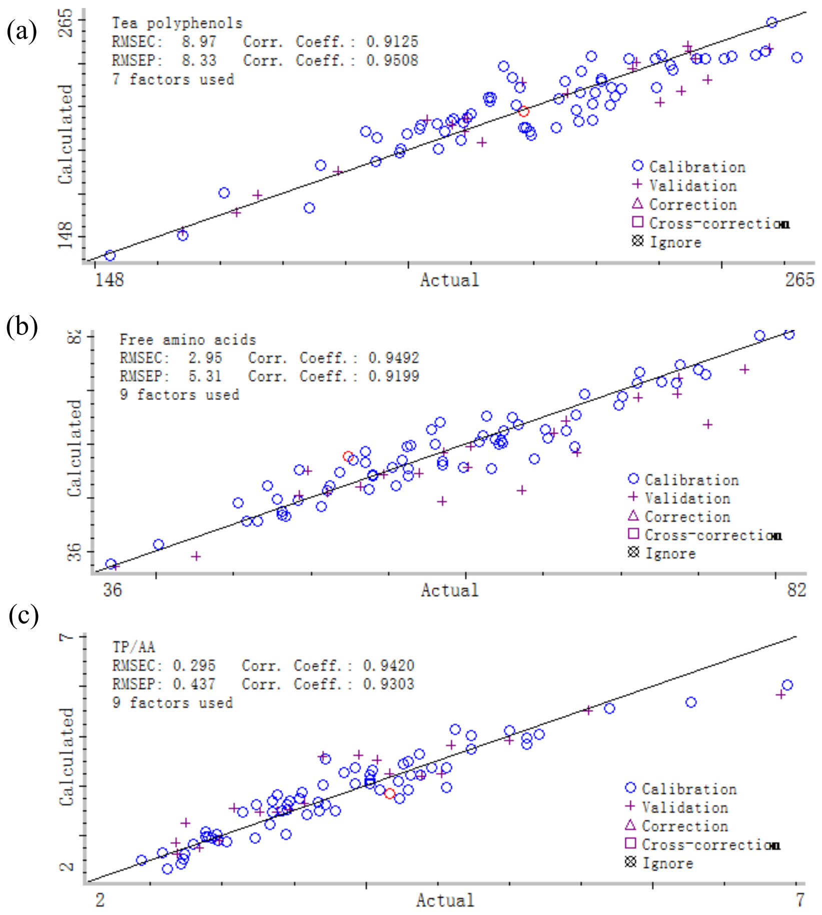

Figure S3. Correlation between the measured and predicted values of biPLS models. (a) TP; (b) FAA; (c) TP/FAA.
